# Astrocytic α_V_β_3_ Integrin Inhibits Neurite Outgrowth and Promotes Retraction of Neuronal Processes by Clustering Thy-1

**DOI:** 10.1371/journal.pone.0034295

**Published:** 2012-03-30

**Authors:** Rodrigo Herrera-Molina, Renato Frischknecht, Horacio Maldonado, Constanze I. Seidenbecher, Eckart D. Gundelfinger, Claudio Hetz, María de la Luz Aylwin, Pascal Schneider, Andrew F. G. Quest, Lisette Leyton

**Affiliations:** 1 Programa de Biología Celular y Molecular, Instituto de Ciencias Biomédicas, Facultad de Medicina, Universidad de Chile, Santiago, Chile; 2 Centro de Estudios Moleculares de la Célula, Instituto de Ciencias Biomédicas, Facultad de Medicina, Universidad de Chile, Santiago, Chile; 3 Programa de Fisiología y Biofísica, Instituto de Ciencias Biomédicas, Facultad de Medicina, Universidad de Chile, Santiago, Chile; 4 Centro de Neurociencias Integradas, Instituto de Ciencias Biomédicas, Facultad de Medicina, Universidad de Chile, Santiago, Chile; 5 Biomedical Neuroscience Institute, Instituto de Ciencias Biomédicas, Facultad de Medicina, Universidad de Chile, Santiago, Chile; 6 Departament of Neurochemistry and Molecular Biology, Leibniz Institute for Neurobiology, Magdeburg, Germany; 7 Department of Biochemistry, University of Lausanne, Epalinges, Switzerland; VIB and KU Leuven, Belgium

## Abstract

Thy-1 is a membrane glycoprotein suggested to stabilize or inhibit growth of neuronal processes. However, its precise function has remained obscure, because its endogenous ligand is unknown. We previously showed that Thy-1 binds directly to α**_V_**β**_3_** integrin *in trans* eliciting responses in astrocytes. Nonetheless, whether α**_V_**β**_3_** integrin might also serve as a Thy-1-ligand triggering a neuronal response has not been explored. Thus, utilizing primary neurons and a neuron-derived cell line CAD, Thy-1-mediated effects of α**_V_**β**_3_** integrin on growth and retraction of neuronal processes were tested. In astrocyte-neuron co-cultures, endogenous α**_V_**β**_3_** integrin restricted neurite outgrowth. Likewise, α**_V_**β**_3_**-Fc was sufficient to suppress neurite extension in Thy-1(+), but not in Thy-1(−) CAD cells. In differentiating primary neurons exposed to α**_V_**β**_3_**-Fc, fewer and shorter dendrites were detected. This effect was abolished by cleavage of Thy-1 from the neuronal surface using phosphoinositide-specific phospholipase C (PI-PLC). Moreover, α**_V_**β**_3_**-Fc also induced retraction of already extended Thy-1(+)-axon-like neurites in differentiated CAD cells as well as of axonal terminals in differentiated primary neurons. Axonal retraction occurred when redistribution and clustering of Thy-1 molecules in the plasma membrane was induced by α**_V_**β**_3_** integrin. Binding of α**_V_**β**_3_**-Fc was detected in Thy-1 clusters during axon retraction of primary neurons. Moreover, α**_V_**β**_3_**-Fc-induced Thy-1 clustering correlated in time and space with redistribution and inactivation of Src kinase. Thus, our data indicates that α**_V_**β**_3_** integrin is a ligand for Thy-1 that upon binding not only restricts the growth of neurites, but also induces retraction of already existing processes by inducing Thy-1 clustering. We propose that these events participate in bi-directional astrocyte-neuron communication relevant to axonal repair after neuronal damage.

## Introduction

Thy-1 is a small, highly conserved, glycosyl phosphatidylinositol (GPI)-anchored surface protein that is present on many cells, such as fibroblasts, ovarian cells, lymphocytes, cancer cells and neurons [1]. In the central nervous system (CNS), high levels of Thy-1 expression are reached during the first postnatal weeks in chicken, rat, mouse, dog, and humans [2], [3]. Despite its conserved and widespread expression, the role of neuronal Thy-1 has remained poorly defined.

Historically, Thy-1 has been suggested to function as an inhibitor of neurite outgrowth *in vitro*. For instance, cells of neural origin, like PC12 and NG115, increase the extension of neuronal-like processes when lacking Thy-1 [4], [5]. Recently, Thy-1-mediated inhibition of neurite outgrowth was suggested to impair neuronal regeneration *in vivo* [6]–[8]. Indeed, a decrease in Thy-1 levels might be required in regeneration of dorsal root ganglion neurons following injury of the sciatic nerve in adult rats [6]. Part of the “scientific facelessness” of Thy-1 stems from the lack of a defined ligand [1]. Hence, studies employing anti-Thy-1 antibodies have prevailed to characterize signaling events triggered by Thy-1.

Anti-Thy-1 antibodies have additionally been used to study changes in Thy-1 mobility on cell membranes and downstream intracellular signaling [9]. Due to its GPI anchor, Thy-1 mobility has been found to be higher than that of transmembrane proteins [10] and highly dynamic changes in lateral diffusion, as well as clustering of Thy-1 in cholesterol-rich micro-domains are thought to trigger Src-dependent downstream signaling events [9], [11], [12]. Interestingly, a mechanism that explains how Thy-1-dependent Src-mediated signaling may be achieved by coupling the events occurring in the outer-leaflet with those in the inner-leaflet of the plasma membrane has been previously proposed [11], [12]. Importantly, such events have not been evaluated using an endogenous ligand for Thy-1.

We have previously shown that endogenous Thy-1, expressed on neuronal cell lines, or recombinant Thy-1-Fc protein, directly binds to α**_V_**β**_3_** integrin *in trans* and triggers a variety of downstream signaling events that lead to focal adhesion and stress fiber formation in DITNC1 astrocytes [13]–[18]. Thus, α**_V_**β**_3_** integrin is a receptor for Thy-1 that induces morphological changes in astrocytes. Although, the potential consequences of Thy-1-α**_V_**β**_3_** integrin interaction for neurons have been suggested [1], [13], [14], [17], [18], these have never been formally shown.

Here, astrocytic α**_V_**β**_3_** integrin was evaluated as a possible ligand for Thy-1 and changes in neurons were assessed. We provide evidence indicating that inhibition of neurite outgrowth is mediated by Thy-1-α**_V_**β**_3_** integrin interaction in neuron-astrocyte co-cultures. Moreover, α**_V_**β**_3_**-Fc triggered retraction of already established neuronal processes and clustering of Thy-1 on neuronal cell membranes. Thy-1 clustering coincided time-wise with a co-distribution of Thy-1 and Src kinase, as well as with increased Src phosphorylation on Tyrosine-527, a marker for kinase inactivation. These observations support a model whereby astrocytic α**_V_**β**_3_** integrin operates as a Thy-1-ligand that triggers neuronal alterations through the engagement of Thy-1. Thus, Thy-1-α**_V_**β**_3_** integrin association represents a novel bidirectional signaling module that connects neurons with astrocytes.

## Materials and Methods

### Cells, peptides and enzymes

CAD cells, semi-adherent immortalized cells derived from cathecolaminergic neurons of mouse CNS, were kindly donated by Dr. Donna Chikaraishi (Duke University Medical Center NC, USA) [19]..The DINTC1 astrocyte cell line was obtained from Dr. Luc Pellerin (University of Lausanne, Switzerland). All cell lines were cultured following reported conditions [17]. Purified primary neurons were derived from brain cortices of 16 day-old rat embryos following published protocols [20] and cultured on poly-L-lysine-coated glass coverslips in 0.5 ml of Neurobasal supplemented with B27, 1% penicillin-streptomycin, and 1 mM glutamine (Gibco). Neurons cultured during 4–5 days were employed for dendrite outgrowth assays. Alternatively, those of 12–15 days were used to study the retraction of neuronal processes and Thy-1 clustering. All procedures used to obtain primary cells were revised and approved by the local Bioethics Committee for Animal Experimentation, Faculty of Medicine, Universidad de Chile (protocol CBA #0259). PI-PLC was purchased from Sigma. Recombinant Fc molecules and their characterization have been previously reported [17].

### Thy-1 knockdown

We generated stable cells with reduced levels of Thy-1 using methods previously described [21], [22] by targeting Thy-1 mRNA with four different shRNA using the lentiviral expression vector pLKO.1 and puromycin selection. Targeted sequences were: shRNA_1_ 5′-CCGCCATGAGAATAACACCAA-3′; shRNA_2_ 5′-CAGCCCTATATCAAGGTCCTT-3′; shRNA_3_ 5′-TGAGAATAACACCAAGGATAA-3′; shRNA_4_ 5′-GTATAGAGACAAGCTGGTCAA-3′. As control, an shRNA against the luciferase gene was employed. Constructs were generated by The Broad Institute (Boston, MA) based on different criteria for shRNA design (see http://www.broadinstitute.org/rnai/trc/lib).

### Morphological differentiation of CAD cells

In all experiments where CAD wild type Thy-1(+) (CAD cells), CAD shRNA Thy-1(−) or CAD shRNA control Thy-1(+) cells were differentiated, the cells were detached by gentle pipetting with 5 ml of PBS, counted and seeded over different substrates for 24 hours in DMEM/F12, 8% FBS, and 1% penicillin-streptomycin. Next day, complete medium was removed, cells were washed with abundant PBS and serum-free medium supplemented with 50 ng/ml SeNa during 1, 24 or 72 hours was added. This procedure reduces the proliferation rate and induces morphological, as well as functional differentiation [19].

### Neurite outgrowth on astrocytes

CAD-DITNC1 co-cultures were employed to study neuron-astrocyte communication *in vitro* [17]. Briefly, DITNC1 astrocytes were grown to 60% confluency on 6-well plastic plates in DMEM/F12, 8% FBS, and 1% penicillin-streptomycin. Then, 8×10^4^ neuronal CAD cells were added to astrocyte monolayers. Next day, cells were washed with PBS and differentiation of CAD cells in serum-free medium was induced for 24 hours. In other experiments, 90% confluent astrocyte monolayers were fixed with 4% p-formaldehyde in 100 mM PIPES pH 6.8, 0.04 M KOH, 2 mM EGTA and 2 mM MgCl_2_ for 15 minutes at room temperature and washed with abundant PBS. Then, fixed monolayers were incubated with Thy-1-Fc or TRAIL-R2-Fc recombinant proteins (600 ng/ml) or with anti-β**_3_** integrin (clone F11, Pharmingen) or anti-β**_1_** integrin (clone Ha2/5, Beckton & Dickinson) antibodies (5 µg/ml) during 1 hour. Next, CAD cells were added and differentiated in serum-free medium as mentioned before. Fixed-cell DIC images were acquired by using a Nikon eclipse E600FN microscope with water immersion 40× (1.2 NA) and 60× (1.4 NA) objectives.

### Neurite outgrowth on α_V_β_3_-Fc-coated plates

For each independent experiment, plastic plates were coated with control or α**_V_**β**_3_**-Fc supernatants for 48 hours. After that, each matrix was incubated with PBS supplemented or not with anti-α**_V_** integrin and anti-β**_3_** integrin or with the equivalent amount of anti-β**_1_** integrin antibodies at room temperature for 15 minutes. Then, solutions containing antibodies were discarded and the plates were washed twice with PBS. CAD, CAD shRNA Thy-1(−) or CAD shRNA control Thy-1(+) cells were seeded over the different matrices in complete medium overnight. Next, neuronal-like differentiation was induced by serum deprivation for 24 hours. After differentiation, cells were fixed and photographed as indicated before.

### Dendrite outgrowth in presence of soluble α_V_β_3_-Fc

Cortical neurons (4–5 days in culture) were treated daily with α**_V_**β**_3_**-Fc-containing supernatants (1∶10) for 3 days. As a control, α**_V_**β**_3_**-Fc-depleted supernatants, obtained by overnight incubation with 1 mg/ml of Protein A-coated beads at 4°C, was used. Integrin α**_V_**β**_3_**-Fc was no longer detected in depleted-supernatants by Western blot analysis (not shown). As an additional control, α**_V_**β**_3_**-Fc-depleted supernatants containing 8.8 µg/ml of purified TRAIL-R2-Fc [13], [18] were also used. In some cases, neurons were pre-treated with 1 U/ml of PI-PLC following a previously reported protocol [4]. As a control, cells were incubated with heat-inactivated PI-PLC. After the different treatments, neurons were fixed and stained with antibodies to MAP-2. Images were acquired using a Nikon eclipse E600FN microscope with water immersion 60× (1.4 NA) objective.

### Neurite retraction and live cell monitoring

CAD wt (4×10^4^) cells were seeded in 3.5 cm plastic plates in complete medium overnight and subsequently left to differentiate for 24 hours in serum-free medium as indicated before. To initiate live cell monitoring, cell medium was replaced by a solution containing 145 mM NaCl, 5 mM KCl, 2 mM CaCl_2_, 1 mM MgCl_2_, 10 mM glucose and 10 mM HEPES, pH 7.4 and 300 mOsm. Then, control supernatants or α**_V_**β**_3_**-Fc-containing supernatants were added after diluting them 1-to-10 fold. Morphological changes were recorded during 75 minutes. In other experiments, CAD wt (2×10^4^) cells were seeded on glass coverslips in 24-well plates with complete medium and incubated overnight. Afterwards, differentiation was induced for 24 hours and cells were then exposed to control supernatants or α**_V_**β**_3_**-Fc-containing supernatants for 75 minutes. Cells were fixed and stained with anti-Thy-1 antibodies and rhodamine-conjugated phalloidin to visualize polymerized actin.

### Neuronal process retraction and Thy-1 clustering assays

Differentiated cortical neurons, 12–15 days in culture *in vitro*, were treated with supernatants containing α**_V_**β**_3_**-Fc fusion protein or with control α**_V_**β**_3_**-Fc-depleted supernatants supplemented or not with 8.8 µg/ml of TRAIL-R2-Fc fusion protein or with 0.1 µg/ml of Protein A for 0, 15, 30 or 60 minutes (Thy-1 clustering evaluation) or 24 hours (process retraction). After these treatments, neurons were fixed and stained with antibodies against Thy-1.

### Immunostaining

To analyze neurite retraction, CAD cells were fixed with 4% p-formaldehyde in 100 mM PIPES buffer, pH 6.8, containing 0.04 M KOH, 2 mM EGTA, and 2 mM MgCl_2_, permeabilized with 0.1% Triton X-100 in PBS containing 1 mM sodium orthovanadate and aprotinin, leupeptin, and benzamidine (10 µg/ml each) for 10 min, and blocked with 1% bovine serum albumin in PBS for 15 minutes. Then, cells were stained with anti-Thy-1 antibodies (1∶200 clone III5 [17], [23]), which were detected with FITC-conjugated secondary antibody. Rhodamine-conjugated phalloidin (1∶2000) was used to detect F-actin. After washing and mounting the coverslips with Mowiol-2.5% 1,4-Diazabicyclo [2.2.2]octane, fluorophores were visualized in a Zeiss LSM-Pascal 5 confocal microscope. Inhibition of dendrite outgrowth of cortical neurons was analyzed by fixing, permeabilizing with 0.1% Triton X-100, and staining with anti-MAP-2 antibodies (1∶1000) followed by a FITC-conjugated secondary antibody (1∶1000).

To study Thy-1 clustering, cortical neurons were fixed with 4% p-formaldehyde in HBSS buffer (Gibco). Next, neurons were washed with a solution containing 10% bovine fetal serum and 0.1 mM glycine in HBSS four times for 5 minutes. Staining was performed with mouse anti-Thy-1 antibody for 20 minutes (1∶200 clone OX7, Millipore) followed by Alexa 488-conjugated secondary antibody (1∶1000). Under these conditions, fixation-induced membrane permeabilization was minimal (Fig. S1). To study α**_V_**β**_3_**-Fc/Thy-1 co-localization, neurons stained for Thy-1 were then incubated in the presence or the absence of supernatants containing α**_V_**β**_3_**-Fc for 15 minutes, washed well with PBS and fixed with 4% p-formaldehyde in HBSS buffer. Bound α**_V_**β**_3_**-Fc was detected using consecutive incubations with a goat anti-human IgG antibody (1∶1000) and a donkey anti-goat IgG Cy3-conjugated antibody (1∶1000). Fluorescence was visualized by using a Zeiss AXIO Imager A2 microscope with a 63× objective (1.4 NA).

### Western blot of Src kinase

Neurons (4×10^5^) were rinsed once with PBS and scraped into 50 µl of sample buffer supplemented with complete protease inhibitor cocktail (CompleteTM, Boehringer Mannheim, Mannheim, Germany) and phosphatase inhibitors (1 mM Na_3_VO_4_ and 100 mM NaF) at 4°C. Samples were electrophoretically separated in 10% SDS-PAGE gels and transferred to nitrocellulose. The nitrocellulose was incubated with blocking solution [5% w/v nonfat dry milk, 0.1% Tween-20 in Tris-buffer saline (TBS), pH 7.4] at room temperature for 1 hour, prior to the addition of the primary antibodies, anti-Src or anti-phosphoTyrosine-527Src (Cell Signaling) used according to the manufacturer's protocol. The secondary antibody used was goat anti-rabbit IgG conjugated to HRP (1∶2000; Calbiochem). Bands were visualized by chemiluminescence (PerkinElmer, Boston, MA, USA). The nitrocellulose was stripped between probing with different antibodies using 100 mM 2-mercapto-ethanol, 2% w/v SDS in TBS pH 6.9, at 60°C for 30 minutes.

### Quantification of neuronal processes and clusters

Processes of CAD cells were quantified by measuring the soma-to-tip distance using the 2D-measurement tool of IMARIS 5.0 (Bitplane, Switzerland). A cell was considered differentiated, when presenting at least one process longer than or equal to 15 µm. Other criteria used here that characterize differentiated cells, included number and length of processes, as well as number of varicosities. The speed of process extension/retraction was estimated by measuring the initial and final length of a process at different time points: speed = [final–initial length (µm)/time (minutes)]. Here, a negative speed value indicates process retraction. Length and number of dendrites of cortical neurons were estimated by using the semi-automatic tracing tool of *ImageJ* (NeuronJ plug in, NIH).

Thy-1- and F-actin-associated fluorescence intensity in CAD cells was independently quantified from inverted images of each fluorescent channel obtained by using *ImageJ*. Cluster images on cortical neurons were processed by using the derivative method of the *ImageJ* software. This corrective method decreases several optic artifacts and thereby improves detection and quantification of cluster-like signals [24], [25]. After processing, the number, area and 1-bit masks of both Thy-1 and Src clusters were obtained by using the “analyze particles” plug-in. Masks were employed to quantify co-distribution of Thy-1 and Src clusters using OpenView software (written by Dr. Noam Ziv, Haifa, Israel, see [26]).

### Statistical analysis

Results were compared by non-parametric Mann-Whitney analysis. Statistical significance is indicated in each figure. In all figures, n is the number of independently performed experiments.

## Results

### Integrin α_V_β_3_ on the cell surface of astrocytes restricts neurite extension

**Figure 1:**
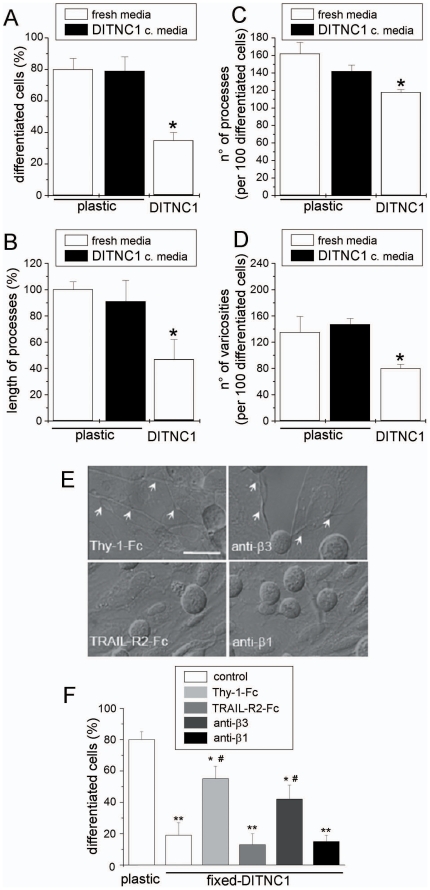
Integrin α_V_β_3_ expressed by DITNC1 astrocytes inhibits neurite extension of CAD cells. (**A–D**) Quantification of four different morphological parameters using IMARIS software (Bitplane, Switzerland) of bright-field microscopy images of CAD cells seeded over plastic, seeded over plastic in serum-free medium previously conditioned by DITNC1 cells for 24 hours (black bars) or over a monolayer of DITNC1 astrocytes in serum-free medium. (**A**) Percentage of differentiated CAD cells with processes ≥15 µm; (**B**) length of the processes extended by differentiated cells, expressed as a percentage of the control over plastic; (**C**) number of processes in 100 differentiated cells and (**D**) number of varicosities per 100 differentiated cells. (**E,F**) CAD cells seeded over fixed-astrocyte monolayers were induced to differentiate. To block α**_V_**β**_3_** integrin, fixed-cells were incubated with recombinant Thy-1-Fc or antibodies against β**_3_** integrin. TRAIL-R2-Fc or antibodies against β**_1_** integrin were used as controls. Co-cultures were photographed (**E**) and the percentage of differentiated CAD cells (**F**) was quantified as in (**A**). Arrows in E indicate axon-like neurites growing over the DITNC1 mololayer. All graphs show mean+s.e.m. determined from at least 100 cells per condition; n = 3. **P<0.01 or *P<0.05 compared with control cells seeded over plastic. #*P*<0.05 compared with their respective control.

The effect of α**_V_**β**_3_** integrin on neurite outgrowth was first examined in cell lines. CAD neuron-like cells were seeded over a monolayer of DITNC1 astrocytes and induced to differentiate by serum deprivation. When seeded on plastic (Fig. 1A), cellular processes of ≥15 µm in length (a sign of morphological differentiation together with length of processes, as well as number of both processes and varicosities) were observed for ∼80% of the cells. In contrast, only ∼35% of the cells developed such extended processes over a monolayer of astrocytes (Fig. 1A). Quantification of the length of processes showed that neurites were ∼55% shorter in CAD cells differentiated over DITNC1 astrocytes (Fig. 1B). Moreover, the number of both processes and varicosities, beadlike swellings of these processes, were decreased by ∼25% and ∼40%, respectively (Fig. 1C,D). Alternatively, serum-free conditioned media obtained from astrocyte cultures did not affect any of the parameters that account for morphological differentiation of CAD cells on plastic (Fig. 1A–D, black bars). Thus, cell-to-cell contact is required for DINCT1 astrocytes to restrict neurite extension of CAD cells.

We next examined whether inhibition of neurite extension was due to binding between neuronal Thy-1 and astrocytic α**_V_**β**_3_** integrin. To this end, differentiation was induced either in the presence of Thy-1-Fc fusion protein or anti-β**_3_** integrin antibodies. To avoid integrin cross-linking and astrocyte responses, astrocyte monolayers were fixed prior to the addition of CAD cells. As negative controls, monolayers were incubated either with TRAIL-R2-Fc fusion protein [13] or with antibodies against β**_1_** integrin. The expression of both α**_V_**β**_3_** and β**_1_** integrins have been previously reported in DITNC1 cells [18]. Neurite outgrowth over plastic in serum-free medium was largely inhibited by fixed-astrocytes both in the absence and the presence of the negative controls (Fig. 1E,F). In contrast, CAD cells readily extended processes on fixed-astrocytes incubated with either Thy-1-Fc fusion protein or anti-β**_3_** integrin antibodies (Fig. 1E,F). To further demonstrate that α**_V_**β**_3_** integrin is responsible for inhibition of neurite outgrowth, CAD cells were differentiated over fixed-DITNC1 cells that had been transfected with siRNA targeting the β**_3_** chain of the integrin. As expected, CAD cells extended neurites on astrocytes lacking the β**_3_** chain (Fig. S2). Thus, α**_V_**β**_3_** integrin present on the surface of DINTC1 astrocytes inhibits neurite outgrowth of CAD cells, probably through interaction with Thy-1.

### α_V_β_3_-Fc effect on neurite extension depends on Thy-1 expression in CAD cells

**Figure 2:**
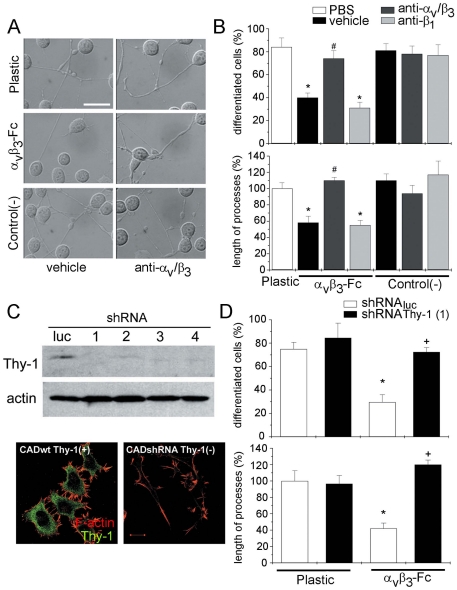
Recombinant α_V_β_3_-Fc is sufficient to inhibit neurite extension of wild type CAD cells but not of CAD Thy-1(−) cells. (**A**, **B**) CAD cells were seeded over plastic, plates pre-coated with α**_V_**β**_3_**-Fc fusion protein or with control supernatants. Where indicated, antibodies against α**_V_** and β**_3_** chains were added. (**A**) DIC pictures obtained 24 hours after inducing process extension over the different substrates. (**B**) Images were analyzed using IMARIS software. The percentage of differentiated CAD cells (upper panel) and the length of processes extended by these cells (lower panel) are shown. Quantification of additional controls, such as antibody vehicle and anti-β**_1_** antibodies are also shown. (**C**) CAD cells lacking Thy-1, generated with Thy-1-targeted shRNA (1–4) and the corresponding control (Luc), were assessed by Western blotting. Actin was used as loading control (upper panels). Thy-1 silencing was further characterized by immunofluorecence (lower panels) staining for Thy-1 (green) and polymerized actin (red). Bar = 15 µm. (**D**) CAD cells lacking Thy-1 or control cells were seeded over plastic or over α**_V_**β**_3_**-Fc-coated plates. Neurite extension was quantified as in (**B**). Graphs show mean+s.e.m. determined from at least 100 cells per condition; n = 3. *P<0.05 compared with respective control cells seeded over plastic. #*P*<0.05 compared with CAD cells seeded on α**_V_**β**_3_**-Fc pre-treated with antibodies against β**_1_** subunit. +*P*<0.05 CAD Thy-1(−) compared with shRNA control cells seeded on α**_V_**β**_3_**-Fc.

To further study the possible involvement of α**_V_**β**_3_** integrin in the inhibition of neurite outgrowth, CAD cells were seeded on α**_V_**β**_3_**-Fc-coated plastic plates. Compared with cells differentiated on plastic or supernatant-coated control plates, neurite extension for CAD cells on α**_V_**β**_3_**-Fc-coated plates was reduced (Fig. 2A, left panels). Such reduction was not observed when the α**_V_**β**_3_**-Fc-coated plates were pre-incubated with anti-α**_V_**β**_3_** integrin antibodies (Fig. 2A, right panels). Quantification of the number of differentiated cells and length of processes revealed that neurite outgrowth was decreased by ∼50%, and the length of processes by ∼40%, in comparison to controls, when CAD cells were seeded on α**_V_**β**_3_**-Fc-coated plates. Additionally, treatment of α**_V_**β**_3_**-Fc-coated plates with antibodies against α**_V_** and β**_3_** integrin, but not β**_1_** integrin, before the addition of CAD cells, eliminated the inhibitory effect of α**_V_**β**_3_**-Fc on neurite extension (Fig. 2B). No such effect was observed for control supernatant-coated plates. Together, these results show that α**_V_**β**_3_**-Fc is sufficient to restrict neurite extension of CAD cells that are serum-deprived.

Evidence indicating direct interaction of Thy-1 with α**_V_**β**_3_** integrin, competitive inhibition of Thy-1-α**_V_**β**_3_** integrin association in CAD-DITNC1 co-cultures, as well as that the astrocyte responses triggered by this ligand-receptor alliance are due to Thy-1-engagement of α**_V_**β**_3_** integrin has been previously reported [13]–[18]. Nevertheless, whether this interaction may be responsible for α**_V_**β**_3_**-mediated inhibition of neurite extension remained to be shown. To test this, we silenced Thy-1 expression by stable delivery of specific shRNA constructs (shRNA Thy-1) using lentiviral vectors. A lentiviral vector containing an shRNA specific for Luciferase was employed as a control (shRNA_luc_ control). Thy-1 expression was evaluated by Western blotting and immunofluorescence analysis. With all four shRNA constructs tested, Thy-1 protein levels were greatly reduced compared to shRNA_luc_ control-transduced cells (Fig. 2C). For immunofluorescence, shRNA Thy-1-transduced cells not only presented almost undetectable Thy-1 levels, but also longer protrusions, consistent with the interpretation that they lack a neurite-outgrowth inhibitor (Fig. 2C). Using both shRNA Thy-1 and shRNA_luc_ control cells, the effect of α**_V_**β**_3_**-Fc-coated plates on differentiation of these cells was evaluated. Similar to wild-type CAD cells (Fig. 2B), morphological differentiation of shRNA_luc_ control cells declined from ∼80% on non-treated plastic to ∼40% on α**_V_**β**_3_**-Fc-coated plates (Fig. 2D). Importantly, shRNA Thy-1 cells were completely resistant to the inhibitory effect of α**_V_**β**_3_**-Fc on neurite outgrowth, and the percentage of differentiated cells and the length of processes were indistinguishable from those of control cells grown on plastic (Fig. 2D). To discard OFF-target effects of the shRNA, CAD cells transduced with different shRNA Thy-1 constructs were also tested. In these cells, α**_V_**β**_3_**-Fc showed no effect on neurite outgrowth (Fig. S3). These results implicate Thy-1-α**_V_**β**_3_** integrin interaction in the inhibition of neurite outgrowth in CAD cells.

### α_V_β_3_-Fc suppresses dendrite extension in primary cortical neurons

**Figure 3:**
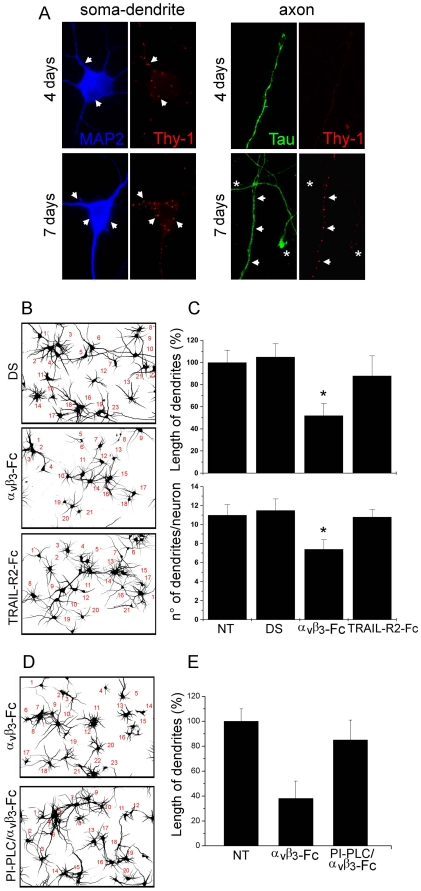
Recombinant α_V_β_3_-Fc inhibits extension of dendrites in cortical neurons. (**A**) Cortical neurons, cultured for 4 and 7 days of culture *in vitro* were stained for Thy-1 (red), MAP-2 (soma/dendrite staining, blue) and Tau (axon staining, green). Arrows indicate some areas with Thy-1 staining. Asterisks label Thy-1-negative axons. (**B–E**) Neurons, cultured for 4–5 days *in vitro* were treated with supernatants containing α**_V_**β**_3_**-Fc fusion protein (**α_V_β_3_-Fc**), α**_V_**β**_3_**-Fc-depleted supernatants (**DS**), α**_V_**β**_3_**-Fc-depleted supernatants supplemented with TRAIL-R2-Fc (**TRAIL-R2-Fc**), or non-treated (**NT**) for 72 hours. Where indicated, 1 U/ml of PI-PLC was added prior to integrin addition (**D,E**). (**B,D**) Inverted fields of MAP-2 fluorescence images that were used to count neurons and evaluate dendrite length. (**C,E**) Quantification of two different morphological parameters performed using Neuro *ImageJ* software. Results shown are the mean+s.e.m. of 720 neurons from three independent experiments (**C**) or 240 neurons from two independent experiments (**E**). *P<0.05 compared with DS condition.

During morphological polarization, Thy-1 expression is restricted to the somato-dendritic compartment in the rodent brain [27]. This was corroborated analyzing Thy-1 presence in soma and dendrites (MAP2-positive), as well as in axons (Tau-positive) of cortical neurons undergoing morphological differentiation after 4 to 7 days in culture *in vitro*. Thy-1 was detected forming clusters at the cell surface of both soma and dendrites after 4 days. Greater number of clusters was observed after 7 days in culture (Fig. 3A). In contrast, there was no detectable Thy-1 signal in growing axons after 4 days (Fig. 3A) or 6 days (not shown). However, after 7 days in culture, a few Thy-1 clusters were observed in axons (Fig. 3A). Coincident with low Thy-1 presence in axons, no changes in axonal growth in the presence of α**_V_**β**_3_** integrin were observed (not shown), Thus, the effect of α**_V_**β**_3_** integrin was further analyzed by evaluating dendrite outgrowth.

After exposing these neurons to α**_V_**β**_3_**-Fc-containing supernatants, dendrites were poorly developed (Fig. 3B, inverted image). Indeed, α**_V_**β**_3_**-Fc-containing supernatant decreased by ∼50% the length of dendrites and by ∼30% the number of dendrites per neuron, compared with non-treated neurons (Fig. 3C). Specificity of α**_V_**β**_3_**-Fc-mediated effects was controlled by using α**_V_**β**_3_**-Fc-depleted supernatants (**DS**), supplemented or not with TRAIL-R2-Fc (**TRAIL-R2-Fc**). Dendrite outgrowth was not affected in neurons exposed to both types of control supernatants (Fig. 3B,C). To further implicate surface Thy-1 in the α**_V_**β**_3_**-Fc-induced inhibition of dendrite outgrowth, GPI-anchored proteins were cleaved from the cell surface by PI-PLC treatment, before the addition of α**_V_**β**_3_**-Fc. PI-PLC treatment prevented α**_V_**β**_3_**-Fc-induced inhibition of dendrite outgrowth (Fig. 3D,E). Taken together, these results suggest that, dendrite extension of primary cortical neurons is reduced in a Thy-1-dependent manner in the presence of the α**_V_**β**_3_** integrin.

### Thy-1 accumulates in collapsed terminals of α_V_β_3_-Fc-treated CAD cells

**Figure 4:**
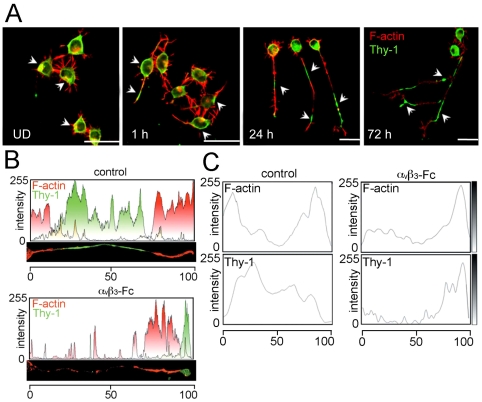
Recombinant α_V_β_3_-Fc induces Thy-1 redistribution and process retraction in differentiated CAD cells. CAD cells were seeded on coverslips in complete media for 12 hours. Differentiation was induced or not (undifferentiated, **UD**), by changing to serum-free medium for 1, 24 or 72 hours. Then, cells were stained for Thy-1 (green, arrowheads) and polymerized actin (red). Samples were visualized by confocal microscopy. Images representative of those obtained in 5 independent experiments are shown. Magnification bars = 20 µm. (**B,C**) CAD cells differentiated for 24 hours were treated with supernatants containing (**α_V_β_3_-Fc**) or not (**control**) α**_V_**β**_3_**-Fc fusion protein. Cells were immunostained for Thy-1 (green) and polymerized actin (red). Fluorescence intensities of one (**B**) or 100 (**C**) neuronal-like processes were quantified by densitometric analysis using *ImageJ* software. Merged colors are shown in yellow (**B**). Average fluorescence intensities of the red (F-actin) and green (Thy-1) staining were obtained for control and α**_V_**β**_3_**-Fc treated cells (**C**). To compare several processes of different lengths, each fluorescence profile was fitted to a 0–100 scale (n = 5).

Thy-1 is reportedly absent in axons during early neuronal development [2], [27]. Later, Thy-1 is highly expressed, but excluded from F-actin-positive segments in stabilized axons of mature neurons [28]. Thus, Thy-1 and F-actin distribution were evaluated in axon-like neurites of differentiated CAD cells by immunofluorescence analysis and confocal microscopy. As reported for primary neurons, Thy-1 was excluded from F-actin-positive segments and its presence was scarce at the tips of CAD cell neurites at 24 and 72 hours of differentiation (Fig. 4A).

Then, CAD cells were employed to study α**_V_**β**_3_**-Fc-mediated effects on Thy-1 localization, since the ultra structure of axon-like processes of differentiated CAD cells resembles that of primary-neuron axons [19]. Thy-1- and F-actin-associated fluorescence was evaluated after 24 hours, because processes were simpler and more reminiscent of axons at this time point in CAD cells. Histograms of fluorescence intensity of separated or merged fluorophores are presented for one axon-like neurite (Fig. 4B) or as the mean of several elongated processes (Fig. 4C). Results obtained with cells treated with control supernatants confirmed that Thy-1 was abundant in segments where F-actin content was low, and vice versa (control panels, Fig. 4B,C). Importantly, Thy-1 was found accumulated in collapsed tips of cells treated with α**_V_**β**_3_**-Fc for 30 minutes (α**_V_**β**_3_**-Fc panels, Fig. 4B,C).

**Figure 5:**
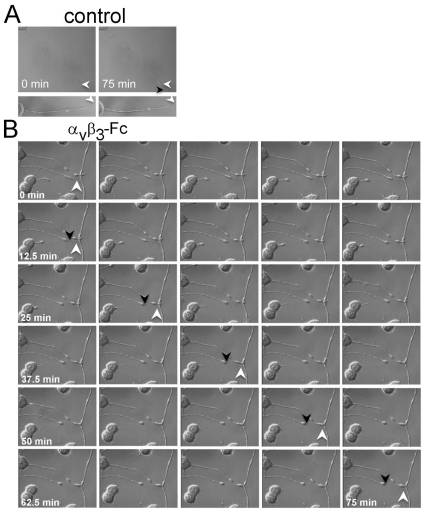
Time course of process retraction induced by recombinant α_V_β_3_-Fc. CAD cells were plated and differentiation was induced as indicated in Fig. 4*A* for 72 hours. Differentiated CAD cells were exposed to (**A**) supernatants depleted of recombinant α**_V_**β**_3_**-Fc (**Control**) or (**B**) supernatants containing α**_V_**β**_3_**-Fc for 75 minutes (**α_V_β_3_-Fc**). Cell morphology was continuously monitored using a digital camera coupled to a microscope with a water-immersion objective and DIC optics. Images captured every 2.5 minutes are shown. White arrowheads indicate the position of the tip of the neurite at time point zero, whereas the black arrowhead shows the end of the trajectory after 75 minutes in (**A**) or after 12.5, 27.5, 42.5, 57.5 and 75 minutes in (**B**). A representative result of 5 independent retraction experiments is shown.

Additionally, terminal collapse preceded rapid neurite retraction, as observed using DIC optic-coupled live cell monitoring photographed during 75 minutes. In control differentiated CAD cells, neurites did not retract during these 75 minutes (Fig. 5A), whereas those exposed to α**_V_**β**_3_**-Fc showed fast and steady retraction (Fig. 5B). The speed of neurite retraction, estimated as indicated in Materials and Methods, was −0.48±0.13 µm/minutes in α**_V_**β**_3_**-Fc-treated CAD cells. To the contrary, those in control condition grew at a speed of 0.02±0.03 µm/minutes. Therefore, α**_V_**β**_3_**-Fc not only induces Thy-1-dependent suppression of neurite outgrowth in CAD cells induced to differentiate, but also triggers redistribution of Thy-1 and axon-like neurite retraction in already differentiated CAD cells.

### α_V_β_3_-Fc-induced Thy-1 retraction of axonal terminals of cortical neurons

**Figure 6:**
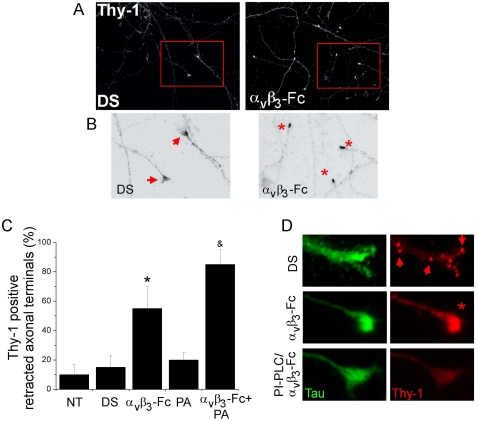
Collapse of axonal terminals of cortical neurons induced by recombinant α_V_β_3_-Fc. (**A, B**) Differentiated cortical neurons, 12–15 days in culture *in vitro*, were treated with supernatants containing α**_V_**β**_3_**-Fc fusion protein (**α_V_β_3_-Fc**) or with α**_V_**β**_3_**-Fc-depleted supernatants (**DS**), supplemented or not with Protein A. Then, neurons were fixed and immunostained for Thy-1 (**A–D**) and Tau protein (**D**) and photographed using conventional fluorescence microscopy. (**B**) Digital zoom was applied to marked areas (red squares in A) and indicated areas were inverted by gray-tone scaling using *ImageJ*. (**C**) Quantification of Thy-1-positive retracted axonal terminals in neurons for each condition is plotted as a percentage of total cells. **NT**, non-treated neurons; **DS**, α**_V_**β**_3_**-Fc-depleted supernatants; **α_V_β_3_-Fc**, α**_V_**β**_3_**-Fc-containing supernatant; **PA**, Protein A; **α_V_β_3_-Fc+PA**, α**_V_**β**_3_**-Fc-containing supernatant supplemented with Protein A (see Methods). Graphs show means+s.e.m. from at least 25 neurons per condition, n = 4. *, &, *P*<0.05 compared to their respective control. (**D**) Representative axonal terminals are shown from neurons treated with α**_V_**β**_3_**-Fc-depleted supernatants (**DS**); α**_V_**β**_3_**-Fc-containing supernatant (**α_V_β_3_-Fc**); or 1 U/ml of PI-PLC added prior addition of α**_V_**β**_3_**-Fc-containing supernatants (**PI-PLC**/**α_V_β_3_-Fc**). Arrows point to Thy-1 clusters in axon and growth cone. Asterisk indicates a retracted axonal terminal.

After morphological polarization of neurons *in vitro*, the axons look thin and uniform in diameter and Thy-1 is mainly present in the axonal-somatic compartment [29]. Thus, we evaluated α**_V_**β**_3_**-Fc-mediated effects on Thy-1 distribution and axon morphology in mature and polarized cortical neurons. Neurons cultured for 12–15 days *in vitro* were treated with α**_V_**β**_3_**-Fc-depleted or α**_V_**β**_3_**-Fc-containing supernatants and Tau-positive/MAP-2 negative staining was used to identify axonal processes (not shown). Typically, a modest but detectable Thy-1 presence was observed in ∼90% of the extended-stabilized axon cones (Fig. 6A,B; DS). Importantly, Thy-1 immune-reactivity accumulated in rounded, retracted tips of neuronal cultures treated with α**_V_**β**_3_**-Fc (Fig. 6A,B; α**_V_**β**_3_**-Fc). Indeed, quantification showed that ∼55% of Thy-1 positive axonal terminals had collapsed in α**_V_**β**_3_**-Fc-treated neurons (Fig. 6C). Thus, in mature cortical neurons, α**_V_**β**_3_**-Fc triggers Thy-1 accumulation at the end of the processes and axonal growth-cone collapse, as similarly observed for axon-like neurites in differentiated CAD cells (Fig. 4).

Based on the absence of a defined ligand, Thy-1 clustering has been artificially induced by using aggregating antibodies in different cell types [9], [11], [30]. Thus, to test the possibility that the α**_V_**β**_3_** integrin may be functionally more effective upon cross-linking, α**_V_**β**_3_**-Fc molecules were bound to Protein A. Indeed, the percentage of axonal terminals that collapsed in the presence of α**_V_**β**_3_**-Fc-Protein A complexes increased to ∼80% (Fig. 6C) suggesting that increased α**_V_**β**_3_** integrin valency favors the axonal retraction process by clustering neuronal receptors.

To confirm Thy-1 involvement in integrin-induced axonal terminal collapse, neurons were treated with PI-PLC before the addition of α**_V_**β**_3_**-Fc and axons were stained with anti-Tau antibodies. As expected, α**_V_**β**_3_**-Fc induced Thy-1 accumulation in the Tau-positive neuronal processes (Fig. 6D). Importantly, even in the presence of α**_V_**β**_3_**-Fc, extended axon terminals were observed in PI-PLC-pretreated neurons with reduced Thy-1 (Fig. 6D), supporting the idea that α**_V_**β**_3_**-Fc effects on growth cone collapse depends on Thy-1 presence.

### α_V_β_3_-Fc-induced Thy-1 clustering on the plasma membrane of neurons

**Figure 7:**
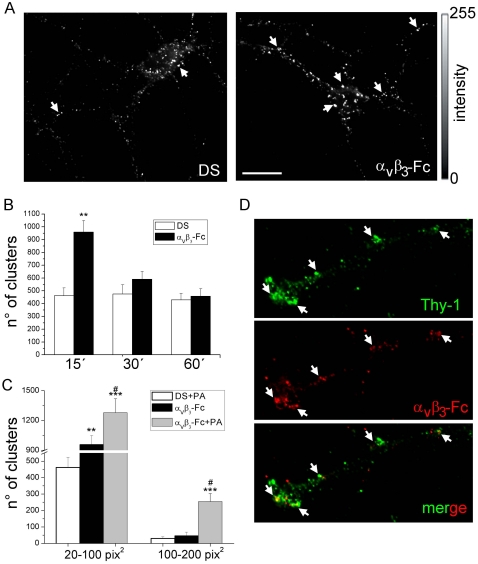
Soluble α_V_β_3_-Fc induces Thy-1 cluster formation on the plasma membrane of living cortical neurons. Mature cortical neurons were treated with supernatants containing α**_V_**β**_3_**-Fc fusion protein (**α_V_β_3_-Fc**) supplemented or not with Protein A (**PA**), or with α**_V_**β**_3_**-Fc-depleted supernatants (**DS**) for 15–60 minutes. Neurons were immunostained for Thy-1 only (**A**) or for Thy-1 and α**_V_**β**_3_**-Fc (**D**). (**A** and **D**) Thy-1 clusters (arrows in **A**) and co-localization with bound α**_V_**β**_3_**-Fc (arrows in **D**) of representative images captured with an epifluorescence microscope are shown. Bar = 20 µm. (**B**) Data plotted as time versus the number of clusters were obtained from images processed using *ImageJ*. Ranges of cluster size from 20 to 100 pix^2^ (1 pix^2^ = 0.01 µm^2^). (**C**) Data plotted as a range of cluster sizes versus the number of clusters for each indicated condition. Results show mean+s.e.m. (12 neurons per condition, n = 6). **P<0.01 and ***P<0.001 compared with their respective control cells in DS at time 15 minutes. *#P*<0.05 between α**_V_**β**_3_**-Fc-Protein A and α**_V_**β**_3_**-Fc (**D**).

Bearing in mind the previous results, the possibility that the α**_V_**β**_3_** integrin may induce clustering of its neuronal receptor Thy-1 was tested. For this purpose, mature neurons were exposed for short periods of time (15–60 minutes) to different supernatants and after fixation, cell surface Thy-1 was identified with antibodies under non-permeabilizing conditions (see Materials and Methods). In non-treated (not shown) or in control neurons treated with depleted supernatants (DS), numerous small clusters were detected on the cell surface (left panel, Fig. 7A). The number and size of these clusters increased significantly in the presence of α**_V_**β**_3_**-Fc for 15 minutes (right panel, Fig. 7A). For processes of neurons exposed to α**_V_**β**_3_**-Fc-depleted supernatants (DS), ∼450 clusters with an area of 20–100 pix^2^ were detected after 15–60 minutes (Fig. 7B). For the same area, the number of clusters increased to ∼950 in the presence of α**_V_**β**_3_**-Fc after 15 minutes (Fig. 7B). The number of clusters decreased after longer treatments (compare α**_V_**β**_3_**-Fc at 15 minutes with 30 and 60 minutes in Fig. 7B). Cumulative distribution of cluster sizes to analyze the frequency of appearance of different clusters was also estimated. However, no changes in cumulative distribution of cluster sizes were observed in neurons treated with α**_V_**β**_3_**-Fc for 15 or 60 minutes (not shown) indicating that α**_V_**β**_3_**-Fc increases the number of a wide-range of Thy-1 clusters. As expected, the number of clusters significantly increased upon incubation with Protein-A (Fig. 7C). Additionally, larger clusters (100–200 pix^2^) were observed in neurons incubated with α**_V_**β**_3_**-Fc-Protein A (Fig. 7C), and the presence of α**_V_**β**_3_**-Fc was detectable in such large Thy-1 clusters (Fig. 7D). Importantly, increased clustering correlated with the axonal terminal retraction found in α**_V_**β**_3_**-Fc- and α**_V_**β**_3_**-Fc-Protein A-treated neurons (Fig. 6). Thus, Thy-1 clustering is likely to represent the first event in the signaling cascade triggered by α**_V_**β_3_ integrin in neurons.

### α_V_β_3_-Fc induces Thy-1-Src co-distribution and Src inhibition in cortical neurons

**Figure 8:**
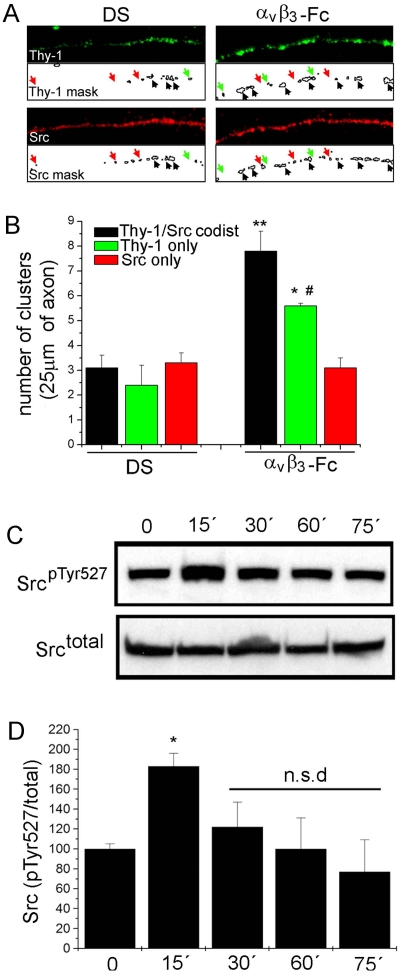
α_V_β_3_-Fc induces Thy-1/Src co-distribution and Src inhibition in cortical neurons. Mature cortical neurons were treated with supernatants containing α**_V_**β**_3_**-Fc fusion protein (**α_V_β_3_-Fc**) or with α**_V_**β**_3_**-Fc-depleted supernatants (**DS**) for 15–75 minutes. (**A**) After 15 minutes, neurons were fixed and immunostained for Thy-1 (green) or total Src kinase (red). Samples were photographed using a conventional fluorescence microscope. Clusters were identified creating a 1-bit mask for each fluorescent channel using *Image J* software. Black arrows indicate Thy-1 clusters co-distributed with Src clusters. Green arrows indicate Thy-1-only, red arrows Src-only clusters. (**B**) Quantification of cluster distribution as indicated. **P<0.01, *P<0.05 compared with number of Thy-1/Src co-distributed clusters in DS. *#P*<0.05 compared to Thy-1/Src co-distributed clusters in α**_V_**β**_3_**-Fc. Results averaged from three independent experiments analyzing 5 axonal segments per experiment are shown. (**C**) At different time points, neurons were lyzed and analyzed by Western blotting. Blots for Src phosphorylated at tyrosine 527 (Src^pTyr527)^ and total Src (Src^total^) are shown. (**D**) Numerical values were obtained by scanning densitometry from three independent experiments and are expressed as phosphorylated/total Src ratio for each time point. *P<0.05 compared to time = 0. n.s.d. indicates non-significant differences compared to time = 0.

Clustering of cell surface Thy-1 with antibodies is known to induce Src kinase recruitment to Thy-1-enriched microdomains at the plasma membrane [30]–[32]. Thus, possible effects of α**_V_**β**_3_**-Fc on Src localization were evaluated by immunofluorescence. After 15 minutes of treatment with α**_V_**β**_3_**-Fc, staining of Src co-distributed with Thy-1 clusters in axons (Fig. 8A). Thy-1-Src cluster co-distribution was analyzed by creating 1-bit masks for both Thy-1 (Thy-1 mask, Fig. 8A) and Src (Src mask, Fig. 8A) fluorescence channels. Co-distribution of Thy-1 and Src clusters increased ∼2.5-fold in integrin-treated neurons compared to control neurons (Fig. 8B). The number of clusters containing Thy-1-only also increased significantly, whereas the number of Src-only clusters did not change with the treatment (Fig. 8B).

Available evidence indicates that clustering of Thy-1 not only recruits Src, but also modifies its activity [9], [31]. To evaluate this possibility, inhibition-associated Src phosphorylation at Tyrosine-527 (Src^pTyr527^) was assessed by immunoblotting. Analysis of Src^pTyr527^ after treatment with α**_V_**β**_3_**-Fc revealed a significant increase after 15 minutes, which decreased thereafter (Fig. 8C,D). Therefore, both Thy-1 clustering and Src inactivation reach their highest values at roughly the same time (∼15 minutes) after stimulation. Additionally, Thy-1 and Src co-distributed in clusters within the plasma membrane, suggesting that all these events might constitute an interconnected signaling cascade triggered by α**_V_**β**_3_** integrin in primary neurons.

## Discussion

Here, we describe a novel function for Thy-1-α**_V_**β**_3_** integrin interaction between neurons and astrocytes. Our studies demonstrate that α**_V_**β**_3_** integrin, present on the astrocyte surface, is sufficient to trigger a Thy-1-mediated functional effect in neurons. We have previously described that the Thy-1-α**_V_**β**_3_** integrin receptor interaction mediates neuron-to-astrocyte communication and signaling events that change astrocyte morphology [17], [18]. Rather intriguingly, α**_V_**β**_3_** integrin is now shown to represent a ligand for Thy-1, which upon interaction triggers dramatic changes in neurons, including inhibition of neurite outgrowth and axonal retraction. Thus, a function induced by an endogenous ligand for this highly abundant glycoprotein is described in neurons. Taken together, results shown here, in conjunction with our previous findings describe a novel mechanism of bidirectional neuron-to-astrocyte communication *in vitro*.

Thy-1 possesses an integrin-binding site (RLD) in its primary sequence that is directly and specifically recognized by α**_V_**β**_3_** integrin receptor in astrocytes [13], [15]–[18]. Evidence supporting the existence of this interaction was obtained using Thy-1(RLD)-Fc, Thy-1(RLE)-Fc, and α**_V_**β**_3_**-Fc recombinant fusion proteins in Surface Plasmon Resonance experiments, as well as using cell-to-cell and cell-to-molecule adhesion assays [13], [15]–[18]. Thus, those and the present findings that include treatment with specific antibodies, competition with recombinant fusion proteins and the use of Thy-1- or β**_3_** integrin-deficient cells, indicate that α**_V_**β**_3_** integrin-induced inhibition of neurite extension is unequivocally dependent on its binding to Thy-1 and signaling events triggered in neurons as a consequence of this interaction.

The presence of a putative ligand for Thy-1 on the cell surface of primary astrocytes maintained in culture during 1–2 months *in vitro* was reported earlier [5], [33]. Indeed, Thy-1(+) but not Thy-1(−) NG115 cells cultured over these long-lasting primary astrocyte cultures, develop less and shorter neurites. Accordingly, our results indicate that DITNC1 astrocytes inhibit neurite outgrowth in a manner that depends on α**_V_**β**_3_** integrin and Thy-1 (Fig. 1 and Fig. S2), and that neurons lacking Thy-1, but not Thy-1(+) cells, grow their processes over α**_V_**β**_3_**-coated plates (Fig. 2D and Fig. S3). Thus, most likely α**_V_**β**_3_** integrin represents an astrocyte surface-bound primary messenger that inhibits the growth of neuronal processes by direct contact with Thy-1.

Establishing Thy-1-mediated signal transduction has been complicated by the fact that it is a GPI-anchored protein that does not span the membrane bilayer. Given its localization in cholesterol-rich micro-domains [11], [12], non-canonical signaling mechanisms, including changes in lateral diffusion and Thy-1 clustering in lipid rafts, are likely to play a role [9], [30]. In both neuronal and non-neuronal cells, changes observed upon cross-linking Thy-1 with antibodies have been associated with signaling via Src family kinases [9], [30], [32], intracellular calcium elevation [34] or shedding of Thy-1 from the plasma membrane [35]. Importantly, α**_V_**β**_3_**-Fc triggered Thy-1 accumulation at the ball-shaped terminals was observed (Fig. 4) suggesting that, upon ligand engagement, Thy-1 redistributes and/or clusters to drive selective retraction of neuritic terminals. Considering that anti-Thy-1 antibodies require 4-fold more time to induce shedding than α**_V_**β**_3_**-Fc to induce redistribution of Thy-1, and that shedding is suggested to stimulate neurite extension [35]; we consider it highly unlikely that α**_V_**β**_3_**-induced Thy-1 shedding was responsible for the observed neurite retraction or inhibition of neurite extension.

Additionally, transmembrane transducer proteins like Csk-binding protein (CBP) are proposed to mediate ligand-induced signaling via Thy-1 in fibroblasts [30]. The transducer serves to connect signaling molecules attached to the outer-leaflet of the plasma membrane with those located in the inner-leaflet of the bilayer [11], [12], such as Src. Our results show that signaling initiated by α**_V_**β**_3_** integrin ligand coincides temporarily with clustering of Thy-1 and Src molecules, as well as with inhibition of Src in living cortical neurons (Figs. 6, 7 and 8). Thus, Thy-1 aggregation is likely to represent the first step of the signaling cascade triggered by α**_V_**β**_3_** integrin, which includes Src inactivation. Interestingly, it has been demonstrated that Src-RhoA GTPase axis is critically involved in the regulation of neurite outgrowth [36]. Currently, studies are underway in our laboratory to address these interesting mechanistic questions.

Thy-1 knockout mice show defects to socially transmit food related cues [37] and a deficiency in synaptic transmission, such as impaired long-term potentiation [38]. Other studies performed with mice carrying a targeted deletion of the Thy-1 gene, show no increase in axon regeneration after two months of a spinal lesion in the corticospinal tract [39]. Nonetheless, given the large number of molecules that inhibit axonal repair, which are present post-injury at the site of lesion, treatments targeting only individual molecules have been modestly successful in achieving functional nerve repair. This has given rise to the notion that combined strategies, which target multiple factors involved in limiting axonal repair are required [40], [41]. Given the abundance of Thy-1 in neurons and the inherent presence of reactive astrocytes in the post-injury zone, the events described here are likely to contribute to inefficient CNS repair. Thus, this Thy-1-α**_V_**β**_3_** integrin pair constitutes a new target for combined therapeutic interventions.

Inhibitory molecules that are up regulated upon injury-induced astrogliosis, not only block initial axonal sprouting, but also promote the formation of clubbed endings, characteristic of axonal retraction [42], [43]. Interestingly, we describe that α**_V_**β**_3_** integrin present in astrocytes induced the retraction of neuritic process terminals (Figs. 5 and 6). Moreover, we also found that Thy-1 localizes to the tip of the axonal terminals upon ligand engagement, likely accounting for its ability to block re-grow and promote retraction of neuronal processes. Thus, consistent with a putative inhibitory role for Thy-1 in regeneration and repair, if α**_V_**β**_3_** integrin is up regulated by astrocytes during astrogliosis, as it has been found in neurodegenerative diseases [44], [45], under ischemia insults [46], [47], or in malignant glioma cells [48], its interaction with the abundantly expressed neuronal Thy-1 could account for α**_V_**β**_3_** integrin-induced axonal retraction and formation of bulbous terminals in injured tissue.

Accordingly, α**_V_**β**_3_** integrin levels are nearly undetectable in late fetal or postnatal astrocytes, in short-term cultures or in mature astrocytes [48]–[51]; thus, the α**_V_**β**_3_** integrin-Thy-1 interaction is not expected to affect neurite outgrowth in adult healthy brains. Therefore, although the enigma of the orphan Thy-1 receptor has been unveiled, the actual *in vivo* role of the α**_V_**β**_3_** integrin-Thy-1 interaction in brain health and disease remains an important issue to be resolved.

In summary, here we show that α**_V_**β**_3_** integrin interacts with Thy-1 in neurons to induce inhibition of neurite extension, as well as retraction of neuronal processes. Thus, evidence for a Thy-1 function triggered by its endogenous ligand is presented here for the first time and agrees with earlier observations suggesting that Thy-1 acts as a receptor for astrocyte-associated ligands leading to inhibition of axonal growth [3], [5].

## Supporting Information

Figure S1 **Immunodetection of Thy-1 clusters on the surface of neuronal plasma membrane.** (**A**) In the left panels, neurons were cultured for 21 days *in vitro* fixed with 4% of p-formaldehyde at room temperature (1), stained with mouse (ms) or guinea pig (gp) anti-MAP-2 primary antibodies, followed by incubation with a Cy3-conjugated donkey anti-mouse IgG or a FITC-conjugated donkey anti-guinea pig IgG, and photographed (2). In the right panels, cells from the same coverslips were permeabilized with 0,1% Triton X-100 (3), and stained again with anti-MAP-2 antibodies of different origin. Note that without permeabilization, antibody diffusion into the neurons was minimal. (**B**) Live neurons were treated with 1 U/ml of PI-PLC and, after fixing and permeabilizing, stained for Thy-1 and MAP-2. Top panels show neurons treated with heat-inactivated PI-PLC (NT); bottom panels, neurons treated with PI-PLC (+PI-PLC). After PI-PLC-treatment Thy-1 clusters are no longer detected on the neuronal plasma membrane, whereas MAP-2 was clearly visible.
(TIF)

Figure S2 **Silencing of β_3_ integrin in DITNC1 cells allows CAD cell differentiation over DITNC1 monolayer.** DITNC1 astrocytes were transfected with 3 different pre-designed siRNA for β**_3_** integrin (500 nM, Ambion), 5′→3′ GCUACUCAUCACCAUUCAU GGUGGAGGAUUACCUGUA, GGAGCAAUCUUUCACUAUU, using siPORT Amine (Ambion). (**A**) Silencing of β**_3_** integrin was evaluated by Western Blot analysis with anti-β**_3_** integrin antibody (Abcam). Scrambled siRNA (Santa Cruz, Biotechnology) was used as a negative control. Actin was evaluated to test sample loading. (**B**) After 48 hours of transfection, 90% confluent astrocyte monolayers were fixed with 4% p-formaldehyde in 100 mM PIPES pH 6.8, 0.04 M KOH, 2 mM EGTA and 2 mM MgCl2 for 15 minutes at room temperature. Then, 8×10^4^ neuronal CAD cells were added to fixed-astrocyte monolayers. Next day, cells were washed with PBS and differentiation of CAD cells in serum-free medium was induced for 24 hours. Quantification of differentiated cells was performed using the plug in *NeuroJ* from *ImageJ* software. Results shown are the mean+s.e.m. (100 neurons per condition, n = 3). **P<0.01 compared with control condition.
(TIF)

Figure S3 **Transduction with different shRNA constructs targeting Thy-1 produces resistance to α_V_β_3_-Fc-induced neurite outgrowth inhibition in CAD cells.** CAD cells were transduced using lentivirus containing shRNA_2_, shRNA_4_ or control shRNA_luc_ as described for Fig. 2C. Then, transduced cells were plated on α**_V_**β**_3_**-Fc-coated or control plates and tested for neurite outgrowth after 24 hours. (**A**) Representative images of each condition are shown (**B**) Length of processes was quantified as mentioned in Material and Methods. Data are mean+SD of two independent experiments (∼80 cells per condition).
(TIF)
