# Supplementary figures and images for: Astrocytic αVβ3 Integrin Inhibits Neurite Outgrowth and Promotes Retraction of Neuronal Processes by Clustering Thy-1
Source: PLoS One. 2012 Mar 30;7(3):e34295. doi: 10.1371/journal.pone.0034295 (PMC3316703; doi:10.1371/journal.pone.0034295)

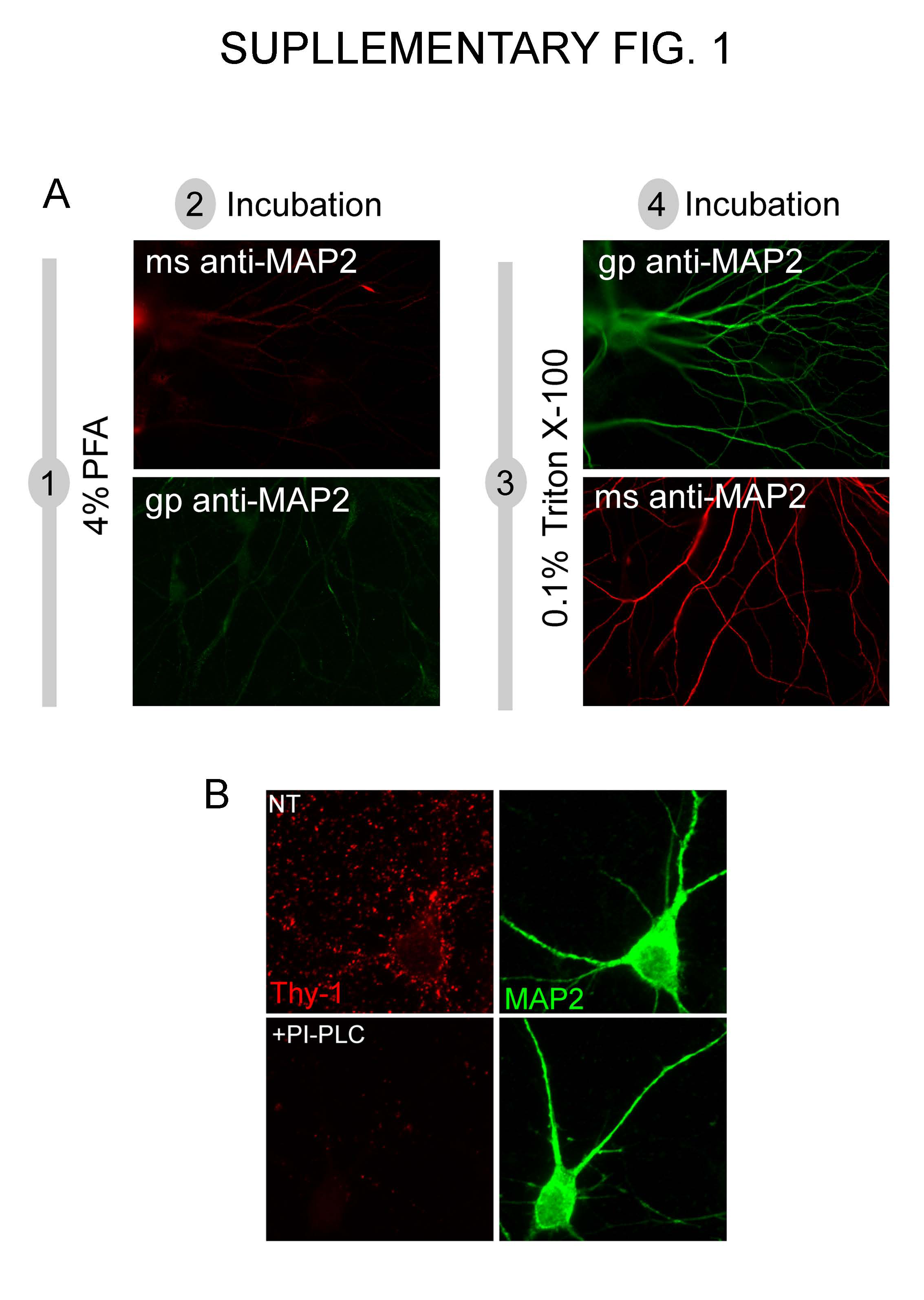

Supplement: Figure S1 — Immunodetection of Thy-1 clusters on the surface of neuronal plasma membrane. (A) In the left panels, neurons were cultured for 21 days in vitro fixed with 4% of p-formaldehyde at room temperature (1), stained with mouse (ms) or guinea pig (gp) anti-MAP-2 primary antibodies, followed by incubation with a Cy3-conjugated donkey anti-mouse IgG or a FITC-conjugated donkey anti-guinea pig IgG, and photographed (2). In the right panels, cells from the same coverslips were permeabilized with 0,1% Triton X-100 (3), and stained again with anti-MAP-2 antibodies of different origin. Note that without permeabilization, antibody diffusion into the neurons was minimal. (B) Live neurons were treated with 1 U/ml of PI-PLC and, after fixing and permeabilizing, stained for Thy-1 and MAP-2. Top panels show neurons treated with heat-inactivated PI-PLC (NT); bottom panels, neurons treated with PI-PLC (+PI-PLC). After PI-PLC-treatment Thy-1 clusters are no longer detected on the neuronal plasma membrane, whereas MAP-2 was clearly visible. (TIF) [file pone.0034295.s001.tif]

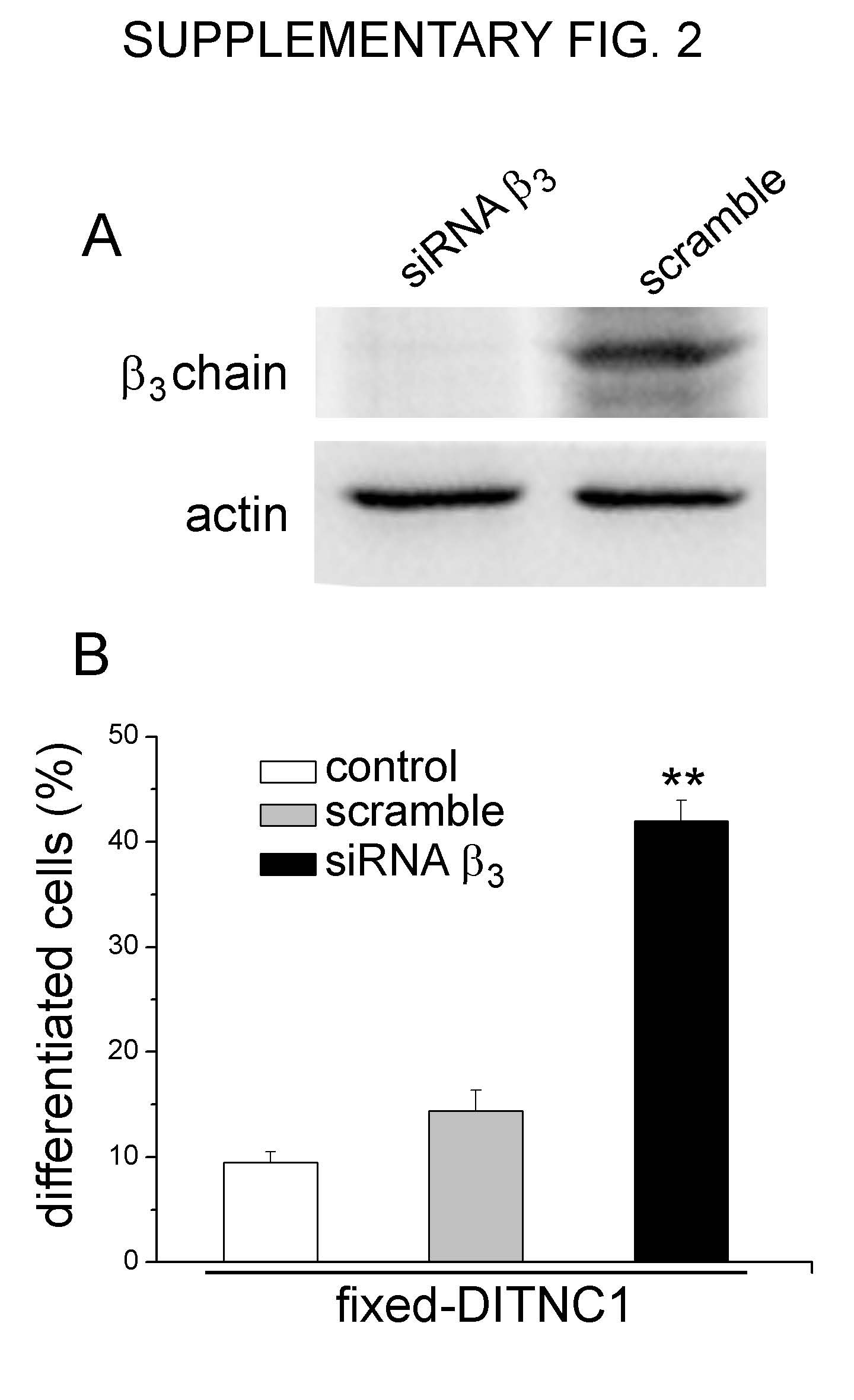

Supplement: Figure S2 — Silencing of β3 integrin in DITNC1 cells allows CAD cell differentiation over DITNC1 monolayer. DITNC1 astrocytes were transfected with 3 different pre-designed siRNA for β3 integrin (500 nM, Ambion), 5′→3′ GCUACUCAUCACCAUUCAU GGUGGAGGAUUACCUGUA, GGAGCAAUCUUUCACUAUU, using siPORT Amine (Ambion). (A) Silencing of β3 integrin was evaluated by Western Blot analysis with anti-β3 integrin antibody (Abcam). Scrambled siRNA (Santa Cruz, Biotechnology) was used as a negative control. Actin was evaluated to test sample loading. (B) After 48 hours of transfection, 90% confluent astrocyte monolayers were fixed with 4% p-formaldehyde in 100 mM PIPES pH 6.8, 0.04 M KOH, 2 mM EGTA and 2 mM MgCl2 for 15 minutes at room temperature. Then, 8×10^4^ neuronal CAD cells were added to fixed-astrocyte monolayers. Next day, cells were washed with PBS and differentiation of CAD cells in serum-free medium was induced for 24 hours. Quantification of differentiated cells was performed using the plug in NeuroJ from ImageJ software. Results shown are the mean+s.e.m. (100 neurons per condition, n = 3). **P<0.01 compared with control condition. (TIF) [file pone.0034295.s002.tif]

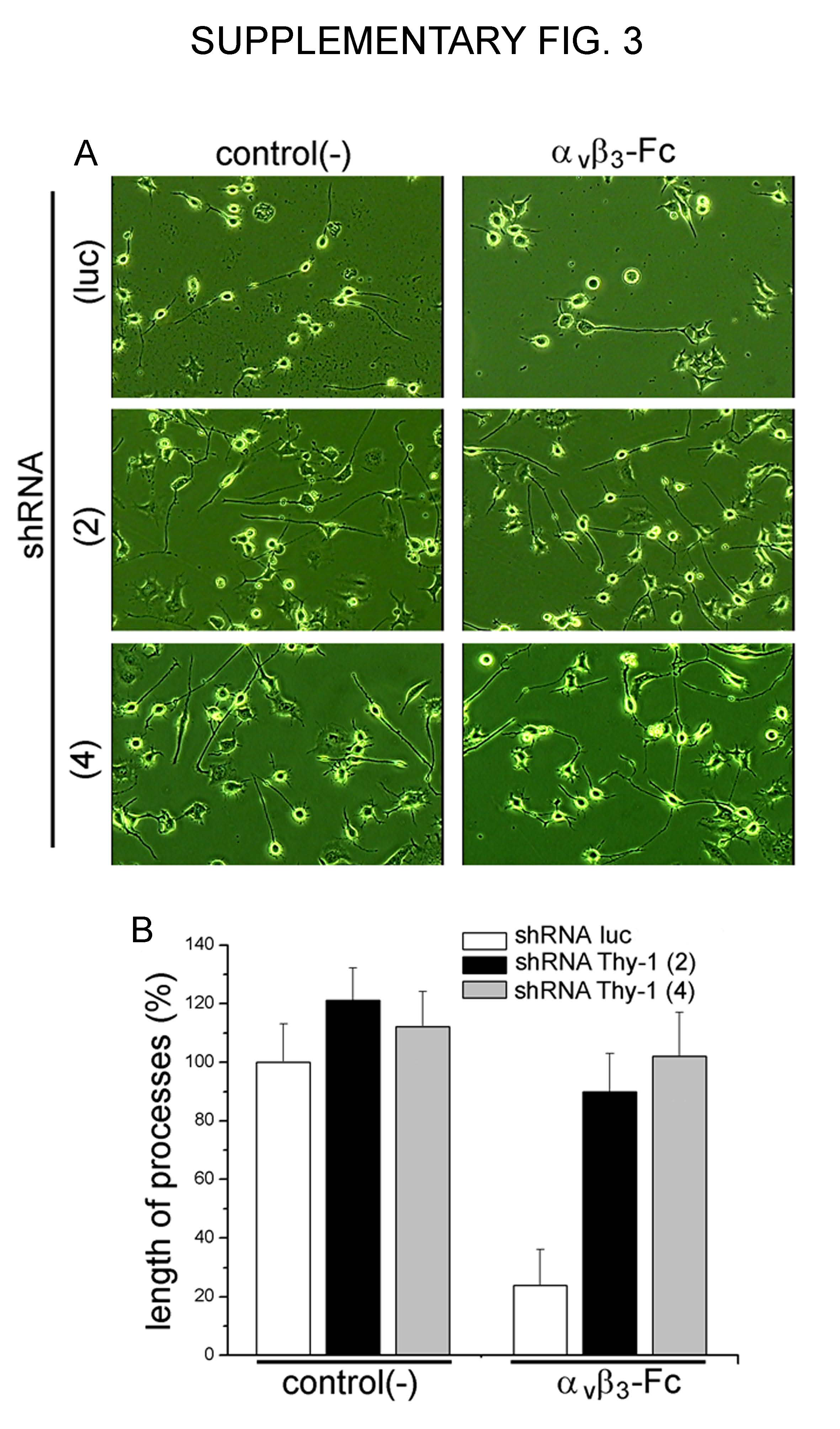

Supplement: Figure S3 — Transduction with different shRNA constructs targeting Thy-1 produces resistance to αVβ3-Fc-induced neurite outgrowth inhibition in CAD cells. CAD cells were transduced using lentivirus containing shRNA2, shRNA4 or control shRNAluc as described for Fig. 2C. Then, transduced cells were plated on αVβ3-Fc-coated or control plates and tested for neurite outgrowth after 24 hours. (A) Representative images of each condition are shown (B) Length of processes was quantified as mentioned in Material and Methods. Data are mean+SD of two independent experiments (∼80 cells per condition). (TIF) [file pone.0034295.s003.tif]
